# Not all depression is created equal: sex interacts with disease to precipitate depression

**DOI:** 10.1186/2042-6410-4-8

**Published:** 2013-04-17

**Authors:** Christina L Nemeth, Constance S Harrell, Kevin D Beck, Gretchen N Neigh

**Affiliations:** 1 Emory University Department of Physiology, Atlanta, GA, USA; 2 VA New Jersey Health Care System, East Orange, NJ, USA; 3 UMDNJ – New Jersey Medical School, Department of Neurology & Neurosciences, Newark, NJ, USA; 4 Department of Psychiatry & Behavioral Sciences, Emory University, 615 Michael Street, Suite 600, Atlanta, GA, 30340, USA

**Keywords:** Depression, Sex difference, Cancer, Epilepsy, Alzheimer’s, Cardiovascular

## Abstract

Depression is a common mental disorder that co-occurs in other neurological and somatic diseases. Further, sex differences exist in the prevalence rates of many of these diseases, as well as within non-disease associated depression. In this review, the case is made for needing a better recognition of the source of the symptoms of depression with respect to the sex of the individual; in that, some disease states, which includes the neuroendocrine and immune reactions to the underlying pathophysiology of the disease, may initiate depressive symptoms more often in one sex over the other. The diseases specifically addressed to make this argument are: epilepsy, Alzheimer’s disease, cancer, and cardiovascular disease. For each of these conditions, a review of the following are presented: prevalence rates of the conditions within each sex, prevalence rates of depressive symptoms within the conditions, identified relationships to gonadal hormones, and possible interactions between gonadal hormones, adrenal hormones, and immune signaling. Conclusions are drawn suggesting that an evaluation of the root causes for depressive symptoms in patients with these conditions is necessary, as the underlying mechanisms for eliciting the depressive symptoms may be qualitatively different across the four diseases discussed. This review attempts to identify and understand the mechanisms of depression associated with these diseases, in the context of the known sex differences in the disease prevalence and its age of onset. Hence, more extensive, sex-specific model systems are warranted that utilize these disease states to elicit depressive symptoms in order to create more focused, efficient, and sex-specific treatments for patients suffering from these diseases and concurrent depressive symptoms.

## Review

Depression is the leading cause of disability in the United States for individuals 15–44 years of age [1]. In addition, depression is predictive of suicide [2], and suicide accounts for more deaths each year than auto accidents [3]. Despite the high incidence of depression and the increased risk of death in depressed individuals, our understanding of the causes of depression is incomplete, limiting the efficacy of available treatments. A more complete appreciation for the range of possible neural substrates that underlie depression may improve our understanding and thereby our treatment of the disorder. Although the phenotype of depression is somewhat consistent, the mechanisms that account for the manifestation of that phenotype are varied. By analogy, one could travel from North America to Australia by many different routes and by many different methods, but once the journey was complete, the view upon arrival would be the same. In the case of depression, the mechanisms that mediate depression in a young woman with a history of chronic abuse and a recent psychological trauma, are likely to differ from the mechanisms mediating depression in an elderly man who recently had a stroke but who has no history of trauma or abuse. While the mechanisms mediating the depression may differ, the core mental and behavioral symptoms for these two individuals could be nearly identical. Two of the most obvious trends that suggest that depression is not a singular entity are the pervasive sex difference in the incidence of depression and the interactions among depression and diseases.

Women are at a two-fold greater risk of developing depression compared to men [4,5] and though it is well established that the prevalence, incidence, and morbidity risk for females is greater throughout life, the underlying mechanisms of this sex difference are relatively undefined. Women experience a steep increase in rates of depression beginning at mid-puberty [5,6]; similarly, women experience fluctuations of sex steroids and corresponding vulnerabilities to mood disorders during and after pregnancy and again at menopause [4]. The pattern of hormonal change parallel to the biological susceptibility to the development of mood disorders in females paints a dramatic role for sex steroids in depression. Despite this relationship, other variables implicated in the pathogenesis of depression, including inflammation [7], socio-demographic demands [5], and a multi-factorial genetic predisposition [4], likely interact with sex steroids to influence specific disease manifestation across individuals. Further highlighting the role of sex steroids in disease, several common medical disorders are exacerbated by hormonal changes associated with the menstrual cycle [8], vulnerabilities that are thought to be related to estrogen’s interactions with both the immune and neuroendocrine systems.

Both neural (e.g. epilepsy [9] and Alzheimer’s disease [10]) and somatic disease (e.g. cancer [11] and cardiovascular disease [12]) are accompanied by a greater prevalence of comorbid mood disorders (see Table 1). The lifetime prevalence of depression among those diagnosed with epilepsy may be as high as 55% depending on the type and severity of epilepsy [9,13]. Pre-existing depression among patients with new-onset epilepsy is up to seven times more common than in a control population (reviewed in Kanner [14]). Depressive episodes in life confer an increased risk of developing dementia or Alzheimer’s disease later in life, while depression is prevalent in 35-50% of patients already diagnosed with Alzheimer’s disease [10]. Among cancer patients, the prevalence of major depression can be greater than 40% depending on the method of diagnosis and type of cancer [11]. Similarly, the prevalence of major depression among cardiac patients is three-fold higher than that observed in the general population [12], and, indicating the bidirectionality of the comorbidity, patients with depression are at a greater risk for a cardiac event in a dose–response manner.

**Table 1:** Depression in disease states: sex differences

**Disease**	**Baseline sex difference**	**Comorbid depression:**	**Disease, sex, & depression:**	**Ref**
Epilepsy	M > F	Between 6-55%; suicide 5-25 higher; depression most associated with temporal lobe epilepsy (TLE); bidirectionality	No sex difference; possibly M > F in left TLE associated depression	[9,13-15,17,19-22,30]
Alzheimer’s disease	F > M (but M > F for morbidity)	35-50%; depressive symptoms in 86%; bidirectionality	History of depression greater risk factor for AD in men; female mice more susceptible to social disinhibition	[10,36,38-42]
Cancer	Type dependent: Liver: M > F Lung: M > F (but F > M given same risk factors) Thyroid: F > M	Up to 40%, depending on type of cancer and severity; bidirectionality	Type and severity dependent; if stratify by severity, sex difference (F > M) is diminished	[11,50,53,72-76]
Cardiovascular disease	Age dependent: M > F younger than 45; F > M older than 45	15-20%; 1/3 of stroke patients; bidirectionality	F > M; approximately twice as high in females or an equivalent ratio to the baseline depressed population	[4,12,86,87,89,90]

From a distance, the interaction between disease and depressive disorders confers no pattern of protection or susceptibility in terms of sex steroids; rather, the unique properties of the disease states in conjunction with sex steroids interact to modulate disease symptomology, progression, and mortality. In this review, we will discuss how sex differences observed within depressive disorders vary in the context of concomitant disease and explore how sex steroids may differentially modulate depression within disease.

### Depression and neural disease

#### Epilepsy, sex, and depression

Epilepsy, or chronically recurring unprovoked cortical seizures, affects approximately two million Americans, and about 140,000 new cases of epilepsy are diagnosed in the United States each year [15]. Over two-thirds of epileptic seizures begin in childhood, and incidence levels in adulthood increase again beginning at age 60 [16]. According to a recent meta-analysis [17], females may have a slightly lower susceptibility to epilepsy than males (46.2 vs. 50.7 per 100,000), though the greater incidence in men may be, in part, attributed to the higher number of risk factors in men, including head injury and stroke. When only idiopathic seizures were considered, however, the sex difference remained [17].

The relationship between depression and epilepsy is not new; Hippocrates himself has been attributed with noting this bidirectionality: “Melancholics ordinarily become epileptics, and epileptics melancholics: what determines the preference is the direction the malady takes; if it bears upon the body, epilepsy, if upon the intelligence, melancholy” (translated and cited in Kanner, 2003 and Mula, 2012 [9,18]). The lifetime prevalence of depression among those diagnosed with epilepsy is 6-55% depending on the type and severity of epilepsy [9,13], and the rate of suicide among epileptic patients may be five to 25 times higher than that of the general population [19] (see Table 1). Conversely, a pre-existing diagnosis of depression among patients with new-onset epilepsy is up to seven times more common than in a control population [14]. Left temporal lobe epilepsy is commonly associated with the greatest propensity for depression [20-22]; however, some have argued that bilateral epilepsy may be somewhat protective against depressive symptoms as reported on the Beck Depressive Inventory [20] and others have reported no difference in laterality and depression [23]. Most depression characterized in epilepsy is interictal, occurring between seizure bouts, though postictal depression, defined explicitly as a depressive mood in the 72 hours following a seizure, has also been identified in a large portion of epileptic patients [9].

Animal models recapitulate the increased susceptibility to depressive-like behaviors in both status epilepticus [24] and absence models [25,26] and may offer insight into the mechanism underlying the association between epilepsy and depression. Mazarati and colleagues [24] showed increased immobility in the forced swim test and decreased saccharin consumption after lithium and pilocarpine-induced status epilepticus, coexisting with decreased hippocampal serotonin and decreased serotonergic release from the dorsal raphe, all indicative of a depressive-like phenotype. Intriguingly, treatment with the selective serotonin reuptake inhibitor, fluoxetine, decreased brain excitability associated with status epilepticus but did not alter performance in the forced swim test [24]. Conversely, the genetically epilepsy prone rat (GEPR) has been shown to be susceptible to the proconvulsant activity of antidepressants, as have cats and rabbits at supratherapeutic doses of these drugs (reviewed in Jobe and Browning [27]). Moreover, the WAG/Rij rat strain is used as a model of absence epilepsy and shows increased immobility in the forced swim test and decreased sucrose consumption, an effect that can be blocked by treatment with the antiepileptic drug, ethosuximide [28]. Russo and colleagues [29] sought to determine if this simultaneously antidepressant and anticonvulsant effect of ethosuximide, a purported calcium channel inhibitor, was specific to that particular drug, which is only effective in absence seizures. Their study revealed that while levetiracetam and zonisamide, other compounds that target calcium channels (but not the sodium-channel targeting carbamezapine), lowered the incidence of spike-wave discharges (SWDs), only ethosuximide lowered SWDs and reduced depressive behavior in the forced swim test [29]. Noradrenergic and serotonergic deficits are believed to cause the seizure predisposition in GEPR and other mammals, which may be linked to depressive vulnerability [27]. Antidepressant anticonvulsant activity may be mediated by the restoration of these deficits; yet, simultaneously, antidepressants can enhance glutamatergic activity and inhibit GABAergic activity, which may lower seizure threshold [27]. These data hint that while depression and epilepsy may share common mechanisms, when combined, the neuropathology is more complex than in a typical depressive pattern alone.

In spite of the two-fold greater prevalence of depression in females in general, several studies have found an absence of a consistent sex difference in epilepsy-associated depression [21,30]. In one large population-based study examining nearly 37,000 people of which 0.6% were diagnosed with epilepsy of heterogenous types, the odds ratio for anxiety disorders or suicidal thoughts among epileptic patients was 2.4 (95% Confidence interval = 1.5-3.8) and 2.2 (1.4-3.3), respectively [30]. Major depressive disorder had a significant age by sex interaction, such that male epileptic patients became more susceptible to depression with age while women became less susceptible [30]. However, this study did not factor in the type of epilepsy, and the greatest sex differences have been seen specifically with left temporal lobe epilepsy (TLE; [20,22]). Left TLE has been associated with a male-specific vulnerability to depression [20,22] as well as more severe depressive symptoms on the Beck Depressive Inventory [20]. This data is particularly interesting, and relevant for clinicians, given the dramatic suicide data among epileptics [19] and the sex disparity in suicide [31]. Collectively, the clinical data points toward a diminished if not altered sex difference in epilepsy-associated depression.

What then causes this alteration of the typical sex difference in depression when associated with epilepsy? It is possible that females simply experience less severe seizures on average. Both in humans and in animal models, progesterone can be anticonvulsant and estrogen proconvulsant, though rats typically experience reductions in seizures during proestrus, when both hormones are at their peak (reviewed in Frye [32]). Estrogen potentiates non-NMDA glutamatergic transmission, but reduces seizures associated with GABA_A_ or NMDA activity [32]. Progesterone’s effects are at least in part due to actions by its metabolite, allopregnanolone, 3α-hydroxy-5α-pregnan-20-one (3α,5α-THP), which is not affected by a menopausal or menstrual state [32,33]. Allopregnanolone is a powerful ligand for the GABA_A_/benzodiazepine receptor complex, which may explain its importance in seizure reduction [32]. Testosterone can provoke proconvulsant activity; castration on the day of birth reduces susceptibility to seizures in the substantia nigra pars reticulata [34], but this effect can be reversed by administration of testosterone [35]. It is possible that while females show a baseline vulnerability to depression, they are somewhat protected from the exacerbation of altered noradrenergic and serotonergic signaling caused by seizure activity due to the effects of ovarian hormones (or their metabolites). Males, on the other hand, may show increased susceptibility, ending up with a final erasure of the sex disparity in depression when associated with epilepsy. This highlights the important fact that depression is mediated by numerous mechanisms – including neurochemical, hormonal, and inflammatory pathways – that may be differentially affected by sex and disease states.

#### Alzheimer’s disease, sex, and depression

Advanced age is the number one risk factor for developing the spectrum of Alzheimer’s disease (AD) related pathologies and risk increases with age [36-38]. Women are at a greater risk of Alzheimer’s pathology [39], although men carry a greater risk of death from the disease [36]. Clinical and pre-clinical studies have shown that females carry an increased risk of developing AD pathology compared to males, even after controlling for increased life span [40,41]. The changing status of sex hormones in aging contributes to the differential diagnoses and prognoses of men and women, as the sudden loss of ovarian hormones following menopause in women is attributed with the increased prevalence of the disease and an observed greater deposition of beta-amyloid (Aβ) plaques in the brains of AD positive women [37]. Depression is diagnosed in 35-50% of Alzheimer’s patients [10], though depressive symptoms are reported in up to 86% of patients [39] (see Table 1). The co-expression of depression with AD is associated with decreased quality of life, more severe cognitive deficits, and decreased longevity [38,39,42] though the diagnosis of depression with AD is a difficult one to make [42]. Depression and AD share similar genetic and environmental susceptibilities [10], which may speak to the bidirectionality of disease expression, yet the underlying mechanisms of the relationship are unclear. Mood disturbances, including depression, are reported to be three to four times as prevalent among the AD population [42], while similarly, a history of mood disorders increases the risk of the development of AD [43]. One study evaluating more than 1,300 patients over 14 years found depression to be a significant risk factor for the development of AD in men, but not women [10], which is an interesting finding in light of the increased rates and pathological load of AD in females. The exact interaction of AD pathology and sex steroids is complex and not well understood, though a role for sex steroids in the epidemiology and pathology of AD is undeniable. Generally speaking, estrogens and androgens are well recognized for their neuroprotective properties, including involvement in promoting cell survival, neuronal differentiation, and reducing inflammation [37,44]. Compared to age-matched controls, reduced circulating levels of 17 beta-estradiol and testosterone are observed in female and male patients with AD, respectively [37,44]. On a cellular level, decreased levels of sex steroids correlate with the increased load of Aβ in both sexes and the increased reactivity of estrogen receptors in women [37]. Again indicative of the dense relationship between the hypothalamic-pituitary-adrenal (HPA) and hypothalamic-pituitary-gonadal (HPG) axes, the HPA axis also alters the relationship between sex and AD. AD patients are reported to exhibit a delayed cortisol recovery following a stressor, and AD patients with higher levels of cortisol perform worse on cognitive tasks [45,46]. Alternatively, age-related mitochondrial dysfunction and consequent increases in reactive oxygen species have been proposed to contribute to the neurodegeneration observed in AD [47].

#### Depression and somatic disease

As discussed above, diseases with direct effects on the brain, such as Alzheimer’s disease, are well documented to cause changes in mental status, including the onset of depressive symptoms, as the disease progresses [48,49]. However, the link between neural diseases and depression is more intuitive than those of non-neuronal diseases. Regardless, connections between peripheral non-neuronal diseases and brain-associated changes paralleling what is observed in depressed subjects are still evident. These studies have identified patient populations with conditions such as peripheral cancers or cardiovascular disease, as well as an incidence of depressive symptoms, and they have then assessed the patient’s brains for neuronal markers of depression. Thus, the changes in neurochemistry and neuroanatomy identified in this way suggest a biological link between these somatic diseases and the abnormal brain activity thought to underlie symptoms of depression.

#### Cancer, sex, and depression

Cancer is the second leading cause of death in the United States after heart disease, and kills more than half a million Americans every year [50]. The top five cancer sites differ in incidence and mortality between men and women but show some overlap: the cancers with the highest incidence in men are, in order, prostate, lung, colorectal, bladder, and melanoma; and those with the highest incidence in women are breast, lung, colorectal, uterine, and thyroid [50]. Lung cancer kills the most men and women each year, and colorectal cancer and pancreatic cancer are also among the top five deadliest cancers for both men and women [50].

Both the cancer disease and the treatments of that disease are documented to cause significant changes in neuroanatomy and neurochemistry. For instance, Cousins and Harper describe a case study where chemotherapy treatment for breast cancer caused a significant reduction in neurotransmitter-related chemistry: myo-inositol, choline and glutamate [51]. Another breast-cancer case study found depressed activation of the anterior cingulate cortex (subgenual cingulate cortex), which was subsequently elevated following behavioral therapy [52]. This provides evidence for both disease and disease-treatment associated effects upon the brain that parallel those observed in depression, and strengthens the proposed association between the occurrence of peripheral cancers and mood disorders.

Depression and cancer often co-exist, with major depression prevalence reportedly greater than 40% depending on the method of diagnosis and type of cancer [11] (see Table 1). Major depression is particularly highly associated with oropharyngeal, pancreatic, breast, and lung cancers, and the prevalence of depression increases with disease severity and progression [11,53]. While the underlying mechanism and the direction of causality in this association remain to be elucidated, several groups have pointed toward HPA axis disruption and immune activation in cancer-related depression. As often seen in non-cancer-related major depression, depressed patients with gynecologic and breast cancer do not suppress cortisol levels after dexamethasone administration [54,55]. Depressed patients with metastatic breast cancer have shown decreased HPA axis response to awakening, and both depressed and non-depressed patients in this study also had a blunted cortisol response to acute stress [56]. In addition, depressed cancer patients display elevated levels of IL-6 relative to both non-depressed cancer patients [55,57] and depressed non-cancer patients [57]. In contrast to this inflammatory state, cancer and stress can also induce immune suppression. Psychological stress, in patients treated surgically for breast cancer, can lower natural killer (NK) cell lysis and response to IFN-γ [58]. As NK cells attack transformed and dying cells without antigen presentation, they may provide an important role in cancer surveillance and thus impaired NK activity is relevant to cancer prognosis [53].

The potential iatrogenic role of cancer treatment to promote immune dysfunction and subsequent depression cannot be ignored. IL-2 and IFN-α are commonly used as cancer treatments and can induce cognitive disturbance, psychological symptoms, and neurovegetative symptoms of depression including loss of appetite, fatigue, and altered sleep [59-61]. Mechanistically, these elevated cytokines may generate glucocorticoid resistance, perhaps by interfering with glucocorticoid receptor translocation [62,63], and thus leading to chronically elevated plasma cortisol associated with the depressive state. Additionally, elevated cytokines can downregulate serotonin [64] and promote indoleamine 2,3 dioxygenase activity, which would further reduce serotonin synthesis by promoting a switch in tryptophan metabolism towards kynurenine and quinolinic acid [65]. This hints at directionality in the link between cancer and depression, suggesting that cancer-associated inflammation promotes neurochemical alterations associated with depression.

While cancer has on the one hand been proposed to promote depressive symptoms by altering HPA and immune function, depression and associated HPA and immune alterations have, on the other hand, themselves been put forth as potential modulators of cancer risk and/or morbidity [53]. Depressed patients are less likely to adhere to medical therapies [66], which may account for some reports of worse outcomes in depressed cancer patients [67]. Sapolsky and Donnelly [68,69] showed that stress-induced growth of a virally-derived tumor is accelerated in aged male rats, potentially due to tumor resistance to glucocorticoid inhibition of glucose transport [69]. In prostate cancer cell lines, glucocorticoids can promote androgen-independent growth [70]. Depression alone has been associated with increased CD4/CD8 ratios, decreased percentages of lymphocytes including T and B cells, and decreased circulating NK cells [71,72]. However, the clinical literature regarding the impact of pre-existing depression on cancer risk and progression remains divided and poorly controlled.

Given the sex-specific susceptibility to certain cancer types, and the bidirectional relationship between cancer and depression, how then does sex affect cancer-related depression? Several studies have attempted to answer this question with mixed results (reviewed in Massie [11]). In a recent Canadian study with over 10,000 patients at two major cancer centers, women were twice as likely as men to report clinical levels of depression as determined using the Psychosocial Screen for Cancer questionnaire [73]. Reported depression varied by cancer type, with the greatest disparity in lung cancer and an absence of a sex difference in neuroendocrine cancers. However, another study examining depression rates using the Hospital Anxiety and Depression Scale in patients with inoperable small cell and non-small cell lung cancer reported that rates of depression in the non-small-cell lung cancer patients differed by sex (31% female vs. 19% male) but not among the small-cell lung cancer patients (46% female vs. 41% male) [74]. Secondary analyses demonstrated that this sex difference or lack thereof correlated with prognosis, such that it existed for the good prognosis patients (with women reporting higher depression) but disappeared among the poor prognosis patients [75]. Miller and colleagues [76] reported a similar finding among patients with stage III or stage IV lung or stage IV gastrointestinal cancer (including colon rectum, pancreas, appendix, and hepatobiliary cancers), using the Beck Depression Inventory II to assess depression. In this study, there were no significant differences between gender and depression or depressive symptom severity [76]. In another study focusing on the role of fatigue, pain, and/or depression in predicting quality of life (QoL) in patients with varying cancer types, women had lower scores for quality of life in the Multidimensional QoL Scale-Cancer and reported greater pain, but equal depressive symptoms on the Center for Epidemiological Studies –Depression Scale [77]. Pain and depression predicted female QoL, while depression alone predicted male QoL, indicating depression as an important factor for both male and female cancer patients. Of course, it is critical to remember that the tools used to assess depression in these studies vary widely, and may account for some disparities seen, making between-study comparisons difficult. However, the trend across assessment tools for a relative leveling of the susceptibility to depression in severe cancer cases seems consistent.

The mechanisms underlying this alteration in sex-specific vulnerability to depression in cancer remain unclear. Some studies have reported increased depression in women associated with estrogen deficiency due to tamoxifen, an estrogen receptor antagonist, or other chemotherapeutic treatment [78,79]. In contrast, one larger study of tamoxifen treatment vs. placebo in non-cancer patients did not find any statistical difference in rates of depression [80,81]. It is however possible that the combination of the cancer stressor combined with the estrogen deficiency may be necessary for induction of the depressive state.

#### Cardiovascular disease, sex, and depression

Aging positively correlates to the incidence and prevalence of cardiovascular-related disease, as age remains one of the greatest risk factors for the emergence of the disease in both men and women [82,83]. Women, however, begin to outnumber men in cardiovascular disease prevalence starting around age 45, and cardiovascular disease is the leading killer of women over the age of 50 [82,84]. Similar to other disease states, the change in demographics of cardiovascular disease and the increased burden on women is attributed to the negative pathologic consequences (loss of antioxidant, anti-inflammatory, and neuroprotective properties) associated with the cessation of ovarian function [85,86].

The manifestation of depression occurs in approximately 15-20% of patients with cardiovascular diseases, a rate three times what is observed in the general population [12] (see Table 1). The links between depression and cardiovascular disease are strong; with evidence supporting the bidirectionality of onset. Low heart rate variability, an indicator of poor autonomic control (i.e. inflexibility), among depressed patients is associated with dramatic effects on vascular function, including increased peripheral resistance, arterial hypertension, and organ damage [87,88]. Depressive events are reported to precede cardiovascular events, and the manifestation of depressive episodes early in life confers a greater risk of the development of cardiovascular disease [4,87]. Similarly, cellular mechanisms associated with mood disorders and stress give rise to vascular damage observed in cardiovascular diseases while the co-expression of the two disease states is associated with the greater risk of cardiovascular events as well as a poorer prognosis following these events [12,87]. Conversely, depression is a common consequence of stroke and other vascular events, occurring in an estimated one third of people [89,90]. Furthermore, patients who have suffered a stroke are at a six-fold greater risk of developing depression for up to two years following the event [91]. Though direct causality is still a mystery, vascular-induced changes to endocrine status [92], neurotransmission [93], and neuroinflammation [94,95] have all been associated with the development of depression.

The linkage of cardiovascular disease to depression, especially late-life depression, is thought to be the product of reduced blood flow [96]. The hippocampus and frontal cortices are susceptible to reduced blood flow and have been shown to exhibit reduced blood flow in depressed patients [97-99]. Moreover, in addition to proposed reduced blood flow, white matter hyperintensities, a sign of hypertension [100], are evident in depression, especially in old age [101,102], and appear to positively correlate with the progress of depressive symptoms [103]. Further, the locations of the white matter intensities correlate with some aspects of the scope of the depressive disorder, though this remains controversial. For instance, when white matter intensities are evident in subcortical areas, a greater percentage of anger attacks are reported in those depressed persons [104]. Similarly, when gray matter hyperintensities have been examined, an apparent positive correlation exists between the presence of those abnormal signals in the basal ganglia and suicide attempts [105], although that study was 85% populated by women.

Sex steroids influence the onset of depressive symptoms as well as the susceptibility to cardiovascular events; however, the interaction of the two disease states is unclear. Though the overall rates of depression are greater among patients with cardiovascular disease, women are still at a double risk of developing depression, a rate that is consistent with what is observed in the general population [12]. Beginning at menopause, a woman’s risk for cardiovascular diseases increases as a result of decreased estrogen activity and parallels a decreased risk for depressive disorders [82,86]. These data suggest that hormonal status can influence mood and cardiovascular disease independently, even when co-expressed. Because men have higher circulating levels of androgens for a greater proportion of their lives, high levels of androgens have often been associated with the development of cardiovascular disease and implicated in the earlier onset of the condition in men relative to women [85,86]. Interestingly, however, circulating levels of testosterone are typically lower in men with cardiovascular disease compared to an age-matched healthy population suggesting either an alternative mechanism or a dynamic role for testosterone [82,86]. Furthermore, low levels of total and bioavailable testosterone in men are associated with depressed mood, although data on the topic are controversial [86,106,107]. The role androgens play in depressive behaviors and cardiovascular disease in men is less clear, and the coexpression of the two disease states does little to elucidate the relationship. In men, other factors may weigh more heavily than androgen status for the emergence of cardiovascular disorders, such as cholesterol [85] and alterations to the HPA axis [84], which both are implicated in depressive disorders [108,109]. Together, these data and others suggest that there is a biological basis of sex-influenced vulnerability for depressive symptoms through a cardiovascular mechanism and that such clinical and preclinical studies should be explored.

## Conclusions

In conclusion, we assert that not all depression is created equal. The evidence presented here indicates that sex interacts with disease to precipitate depression whether the disease is neural (as in epilepsy and AD) or somatic (e.g., cancer and cardiovascular disease) in origin. The relationships described in this review highlight the complex disease-depression interaction as well as the multitude of factors that influence the expression and severity of each condition. In each of the diseases discussed herein, the prevalence of comordbid mood disorders is much higher than in the general population (see Table 1), though in each case the direction of causality is unclear. It appears as though the directionality of the depression-disease relationship is two-fold, such that each state lowers the threshold for the other, depending on the genetic and environmental susceptibilities of each.

The typical 2:1 female predominance in the presentation of mood disorders is tempered in at least some comorbid disease states. Baseline changes in neurochemical signaling due to the disease state, as in epilepsy [32], altered neurobiology secondary to inflammatory mechanisms associated with disease, as in cancer [53,59], or a combination of these factors seem to interact with sex to alter vulnerability to mood disorders. The exact role of sex steroids and whether fluctuations (eg. androgens in cardiovascular disease and estrogen in AD) or absolute levels of hormone may mitigate some degree of disease progression and susceptibility to depression remains unclear. A review by Cahill [110] of “His brain, her brain” similarly addresses the neurobiology of sex differences in disease and advocates for appropriate treatment in light of these differences. Indeed differential coping styles of men and women [111] may contribute at least in part to depressive symptoms; however, several studies have cited not only the bidirectional nature of depression and disease [14,53,87], but the biological basis for depression within disease [95,112,113].

The precise systems that are dysregulated in the manifestation of depression likely vary by the root cause of the disorder. The originating biological cause of depression will depend on the sex, age, and disease state of the individual. For instance, the point of origin is likely to vary substantially between a healthy adolescent girl abused as a child, an adult woman with epilepsy, and an elderly man with cardiovascular disease and a history of cerebral ischemia. Once it is understood that depression is not a singular disorder but rather a common phenotype for a variety of underlying biological disruptions, both central and peripheral in origin, preclinical research will be better designed to identify and target potential therapeutic mechanisms, leading to more specific and effective clinical treatments.

## Abbreviations

3α,5αTHP: 3α-hydroxy-5α-pregnan-20one; Aβ: Amyloid beta; AD: Alzheimer’s disease; GABA: Gamma-Aminobutyric acid; GEPR: Genetically epilepsy prone rat; HPA: Hypothalamic-adrenal-pituitary; HPG: Hypothalamic-adrenal-gonadal; NK: Natural killer; NMDA: N-methyl-D-aspartate; QoL: Quality of Life; SWD: Spike-wave discharge; TLE: Temporal lobe epilepsy.

## Competing interests

CLN declares that she has no competing interests. Authors CSH, KDB, and GNN are members of the Organization for the Study of Sex Differences, for which the *Biology of Sex Differences* is the official journal.

## Authors’ contributions

CLN and CSH participated equally in the literature review, interpretation of the findings, and drafting of the manuscript. KDB helped conceive of the review and participated in drafting. GNN helped conceive of the review, coordinated the efforts of the literature search and interpretation, and participated in drafting the manuscript. All authors read and approved the final manuscript.
